# Three-dimensional structures of *Plasmodium falciparum* spermidine synthase with bound inhibitors suggest new strategies for drug design

**DOI:** 10.1107/S1399004714027011

**Published:** 2015-02-26

**Authors:** Janina Sprenger, Bo Svensson, Jenny Hålander, Jannette Carey, Lo Persson, Salam Al-Karadaghi

**Affiliations:** a Center for Molecular Protein Science, Lund University, SE-221 00 Lund, Sweden; b Department of Experimental Medical Science, Lund University, SE-221 84 Lund, Sweden; c SARomics Biostructures AB, Box 724, SE-220 07 Lund, Sweden; d Chemistry Department, Princeton University, Princeton, New Jersey, USA

**Keywords:** spermidine synthase, *Plasmodium falciparum*, plyamines, structure-based drug design, malaria

## Abstract

The enzymes of the polyamine-biosynthesis pathway have been proposed to be promising drug targets in the treatment of malaria. Spermidine synthase (SpdS; putrescine aminopropyltransferase) catalyzes the transfer of the aminopropyl moiety from decarboxylated *S*-adenosylmethionine to putrescine, leading to the formation of spermidine and 5′-methylthio­adenosine (MTA). In this work, X-ray crystallography was used to examine ligand complexes of SpdS from the malaria parasite *Plasmodium falciparum* (*Pf*SpdS). Five crystal structures were determined of *Pf*SpdS in complex with MTA and the substrate putrescine, with MTA and spermidine, which was obtained as a result of the enzymatic reaction taking place within the crystals, with dcAdoMet and the inhibitor 4-methylaniline, with MTA and 4-aminomethyl­aniline, and with a compound predicted in earlier *in silico* screening to bind to the active site of the enzyme, benzimidazol-(2-yl)pentan-1-amine (BIPA). In contrast to the other inhibitors tested, the complex with BIPA was obtained without any ligand bound to the dcAdoMet-binding site of the enzyme. The complexes with the aniline compounds and BIPA revealed a new mode of ligand binding to *Pf*SpdS. The observed binding mode of the ligands, and the interplay between the two substrate-binding sites and the flexible gatekeeper loop, can be used in the design of new approaches in the search for new inhibitors of SpdS.

## Abstract 2

In this work, X-ray crystallography was used to examine ligand complexes of spermidine synthase from the malaria parasite *Plasmodium falciparum* (*Pf*SpdS).

## 1. Introduction   

Malaria remains a major life-threatening disease, mainly affecting countries in tropical regions. The introduction of artemisinin-based therapies in combination with a diverse range of protective and prevention measures has reduced the number of severe malaria infections in the past decade. However, in 2011 there were still around 216 million clinical cases of malaria, resulting in about 660 000 deaths (World Health Organization, 2012 ▶). Most of these cases were infections caused by the parasite *Plasmodium falciparum*. The fast-evolving drug resistance of this parasite, which is demonstrated by the recent discovery of artemisinin-resistant strains in some Asian regions (Manske *et al.*, 2013 ▶; Miotto *et al.*, 2013 ▶), further emphasizes the urgent need for new, cheap and effective antimalarial therapies.

**Figure 1:**
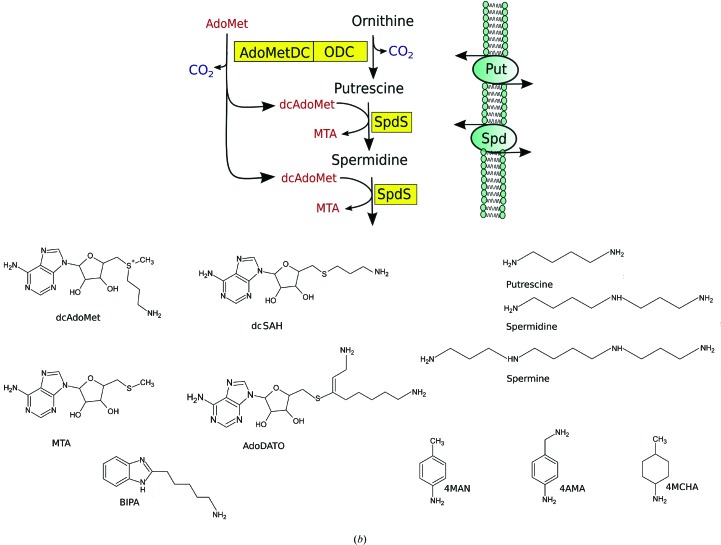
Polyamine biosynthesis. (*a*) Schematic representation of the polyamine-metabolic pathway in *P. falciparum*. (*b*) Chemical structures of the compounds used in the present study.

Polyamines are essential for cell growth and proliferation (Wallace *et al.*, 2003 ▶; Gerner & Meyskens, 2004 ▶; Casero & Marton, 2007 ▶). Cells require well balanced levels of the most abundant polyamines (putrescine, spermidine and spermine), while disturbances in polyamine levels can cause growth arrest or cell death (Persson, 2009 ▶; Nowotarski *et al.*, 2013 ▶). These observations suggested that inhibition of the polyamine-biosynthesis pathway could be used as a strategy for the treatment of cancer and tropical diseases (Bacchi *et al.*, 1980 ▶; Müller *et al.*, 2008 ▶; Birkholtz *et al.*, 2011 ▶). The latter was supported by the success of DFMO (difluoromethyl ornithine) in the treatment of African sleeping sickness caused by *Trypanosoma brucei* (Wickware, 2002 ▶). DFMO inhibits the first step in the polyamine-biosynthesis pathway: the decarboxylation of ornithine catalyzed by ornithine decarboxylase (ODC; Fig. 1 ▶ *a*). Inhibitors of other enzymes of the polyamine-biosynthesis pathway have also been considered for the treatment of tropical diseases (Heby *et al.*, 2007 ▶; Birkholtz *et al.*, 2011 ▶). Among those are *S*-adenosylmethionine decarboxylase (AdoMetDC), which catalyzes the conversion of *S*-adenosylmethionine into decarboxylated *S*-adenosylmethionine (dcAdoMet; Fig. 1 ▶ *b*), and spermidine synthase (SpdS), an aminopropyltransferase which transfers the *N*-aminopropyl group of dcAdoMet to putrescine to form spermidine and 5′-methylthioadenosine (MTA; Fig. 1 ▶ *a*). Another enzyme in this pathway, also a member of the aminopropyl transferase family, is spermine synthase (SpmS), which uses dcAdoMet and spermidine as an aminopropyl donor and acceptor, respectively, to synthesize spermine (Pegg, 2009 ▶; Pegg & Michael, 2010 ▶).

ODC, AdoMetDC and SpdS, but not SpmS, are present in *P. falciparum*, although it has been reported that *Pf*SpdS may use spermidine and dcAdoMet to synthesize spermine (Haider *et al.*, 2005 ▶). This has been corroborated by a crystal structure of *Pf*SpdS containing MTA and spermine in the active site (PDB entry 3b7p; Structural Genomics Consortium, unpublished work). Similarly to human cells, an additional source of polyamines for *P. falciparum* appears to be polyamine import by specific transporters (Ramya *et al.*, 2006 ▶). However, it is currently unclear whether polyamine transport can compensate for depleted internal polyamine levels when polyamine biosynthesis is chemically inhibited in the parasite.

SpdS is one of the best-studied enzymes of the polyamine pathway. Crystal structures of bacterial (*Thermotoga maritima*, *Escherichia coli*, *Pyrococcus horikoshii* and *Helicobacter pylori*), *Trypanosoma cruzi* (PDB entries 3bwb and 3bwc; Structural Genomics of Pathogenic Protozoa Consortium, unpublished work), human, plant (*Arabidopsis thaliana*; PDB entry 1xj5; Center for Eukaryotic Structural Genomics, unpublished work), *Caenorhabditis elegans* and *Pf*SpdS have been determined alone or in complex with various ligands (Korolev *et al.*, 2002 ▶; Dufe *et al.*, 2005 ▶; Lu *et al.*, 2007 ▶; Wu *et al.*, 2007 ▶; Zhou *et al.*, 2010 ▶). The first SpdS X-ray structure to be determined was that from *T. maritima* (Korolev *et al.*, 2002 ▶). This work showed a structure folded into two domains: an N-terminal β-sheet domain (residues 41–97) and a C-terminal Rossmann-fold type domain (residues 98–321). The C-terminal domain has pronounced structural homology to class I methyl transferases that use AdoMet as a methyl donor. The active site of SpdS is located between the domains and consists of two interconnected clefts for binding dcAdoMet and putrescine. A conserved flexible structural element called the gatekeeper loop covers the active site of the enzyme (Korolev *et al.*, 2002 ▶). In the structure of the complex of *T. maritima* SpdS with the inhibitor *S*-adenosyl-1,8-diamino-3-thio-octane (AdoDATO; Fig. 1 ▶ *b* shows the scaffold of the compounds mentioned in the text) the gatekeeper loop had a well ordered structure, while in the ligand-free enzyme it could not be localized in the electron-density map. AdoDATO was designed as a multi-substrate analogue inhibitor and had IC_50_ values of 0.012–0.4 µ*M* (depending on the concentration of dcAdoMet in the assay) for rat SpdS (Tang *et al.*, 1980 ▶; Pegg *et al.*, 1983 ▶) and 0.7 µ*M* for *E. coli* SpdS (Pegg *et al.*, 1983 ▶).

The crystal structure of *Pf*SpdS has been determined in the ligand-free form and in complex with dcAdoMet, MTA and the inhibitors AdoDATO and *trans*-4-methylcyclohexylamine (4MCHA; Dufe *et al.*, 2007 ▶). 4MCHA was designed to occupy the putrescine-binding site and had moderately potent inhibitory activity, with an IC_50_ of 1.7 µ*M* for rat SpdS (Shirahata *et al.*, 1991 ▶). The inhibition of *Pf*SpdS by 4MCHA and AdoDATO has also been studied and revealed IC_50_ values for 4MCHA within the range of the enzymes from other organisms, but somewhat higher IC_50_ values for AdoDATO (1.4 and 8.5 µ*M*, respectively; Haider *et al.*, 2005 ▶; Dufe *et al.*, 2007 ▶). However, on using lower concentrations of dcAdoMet in the assay an IC_50_ of 0.5 µ*M* was obtained for the inhibition of *Pf*SpdS by AdoDATO, a value that is in the same range as those for other organisms (L. Persson, unpublished observation). The structures of the complexes show that AdoDATO binds to *Pf*SpdS in the same way as to *T. maritima* (Korolev *et al.*, 2002 ▶) and that 4MCHA is indeed bound at the putrescine site. In the structures of the complexes with AdoDATO and with dcAdoMet, the gatekeeper loop (residues 196–208 in *Pf*SpdS) is ordered. On the other hand, in the apo structure this loop is completely or mostly disordered (Dufe *et al.*, 2007 ▶). Šečkutė *et al.* (2011 ▶) also demonstrated that the dcAdoMet analogue decarboxylated *S*-adenosylhomocysteine (dcSAH) inhibits human, *T. maritima* and *Pf*SpdS in a dose-dependent manner, with IC_50_ values in the low micromolar range. Interestingly, the IC_50_ value for *Pf*SpdS was about tenfold lower than that for human SpdS. In the crystal structure of human SpdS in complex with dcSAH the inhibitor was shown to bind to the dcAdoMet site with the gatekeeper loop partly ordered (Šečkutė *et al.*, 2011 ▶).

Several other compounds have been used to study the inhibition of SpdS. Shirahata *et al.* (1991 ▶) studied a series of cyclohexylamine-derivative and cyclic aniline-derivative compounds that have also been suggested to bind to the putrescine-binding site of the enzyme. In general, cyclohexylamine-based compounds were shown to be better inhibitors of SpdS. For example, while 4MCHA had an IC_50_ value of 1.7 µ*M*, 4-methylaniline (4MAN) inhibited rat SpdS with an IC_50_ value of 108 µ*M* (Shirahata *et al.*, 1991 ▶). However, despite the low inhibition activity, anilines are less lipophilic than cyclohexylamine-based compounds, are easier to modify chemically and may therefore be better choices for further development into lead compounds. A relatively recent attempt to design SpdS inhibitors used *in silico* screening of compound libraries (Jacobsson *et al.*, 2008 ▶). Of the 2.6 million compounds in the library, 28 hits were selected for binding analysis using NMR spectroscopy. Of these, seven were found to bind to the enzyme and one of them, 5-(1*H*-benzimidazol-2-yl)pentan-1-amine (BIPA; Fig. 1 ▶ *b*), showed strong binding affinity (Jacobsson *et al.*, 2008 ▶). Docking studies predicted the aminopentanyl moiety of this compound to occupy the putrescine-binding site and the benzimidazole part was predicted to occupy the *N*-aminopropyl site. However, crystal structures of SpdS in complex with aniline derivatives or with BIPA have not been reported previously, leaving the details of their mode of interaction with the protein largely unknown.

In the present study, X-ray crystal structures of *Pf*SpdS were solved in complex with MTA and the substrate putrescine, and with the reaction products MTA and spermidine, which was obtained by soaking putrescine into crystals of *Pf*SpdS in complex with dcAdoMet. X-ray crystallography was also used to study the complexes of *Pf*SpdS with BIPA, 4MAN and the amino derivative of 4MAN, 4-aminomethyl­aniline (4AMA). The structures provide new insights into the details of ligand binding to SpdS, the interdependence of the two parts of the enzyme active site and the role of the gatekeeper loop in ligand binding. The results establish experimentally that the putrescine-binding site may accommodate small aromatic compounds and not only aliphatic amines, a finding which, together with the other results, may lead to new approaches for the design of SpdS inhibitors.

## 2. Materials and methods   

### 2.1. Protein expression, purification and crstallization   

For recombinant expression of *Pf*SpdS, the DNA sequence of a 39-residue N-terminally truncated construct in the p15-Tev-LIC vector was used as described previously (Dufe *et al.*, 2007 ▶). Dufe *et al.* (2007 ▶) demonstrated that the truncation of 39 amino acids from the N-terminus of *Pf*SpdS markedly improved both the expression and the crystallization of the protein. Moreover, the values of *K*_m_ for the substrates dcAdoMet and putrescine measured using the 39-residue truncated PfSpds (Lo Persson, unpublished observation) were similar to those reported earlier for a 29-residue N-terminally truncated *Pf*SpdS (Haider *et al.*, 2005 ▶), suggesting that the additional truncation did not affect the enzyme activity. The plasmid produced *Pf*SpdS with six N-terminal His residues and a TEV protease cleavage site. The protein was expressed in *E. coli* BL21 (λDE3) Rosetta Oxford cells. A colony was incubated overnight at 37°C in 20 ml LB medium containing 100 µg ml^−1^ ampicillin. For large-scale expression, 10 ml of the overnight culture was transferred into flasks containing 1 l LB medium with 100 µg ml^−1^ ampicillin and grown at 37°C until the OD_600_ reached 0.5. Expression was induced by the addition of IPTG to 0.5 m*M* for 4–5 h at 37°C. The cells were harvested, lysed and purified as described previously (Dufe *et al.*, 2007 ▶), except that after the Ni-affinity chromatography column gel filtration was used instead of anion-exchange chromatography.

For crystallization, the His-tag was removed using TEV protease. The protease cloned into plasmid BL21pPIPL *E. coli* (Stratagene; a kind gift from Dr H. Berglund) was used for expression of TEV protease and purified as described previously (van der Berg *et al.*, 2006 ▶). *Pf*SpdS (5 mg) was incubated overnight at room temperature with 2 mg TEV protease in 10 ml 50 m*M* NaCl, 10 m*M* HEPES pH 7.5. The digestion mixture was purified using Ni-affinity chromatography followed by gel filtration, using an elution buffer consisting of 500 m*M* NaCl, 100 m*M* HEPES pH 7.5. The protein was concentrated in this buffer to 10–15 mg ml^−1^ and incubated for 30 min at room temperature prior to crystallization with either a threefold molar excess of dcAdoMet or MTA or a fivefold molar excess of BIPA. Protein that had been pre-incubated with either MTA or dcAdoMet was further incubated with a threefold molar excess of 4MAN or 4AMA. Proteins were crystallized using hanging-drop vapour diffusion at 295 K with a reservoir solution consisting of 0.1 *M* MES buffer pH 5.6, 0.1 *M* ammonium sulfate, 27% PEG 3350. The protein–ligand solution was mixed with the reservoir solution in a 1:1 volume ratio to give a total drop volume of 2 µl. Crystals usually appeared after 2 d. Soaking experiments for the crystal structures of MTA with putrescine and to test whether reaction can take place when putrescine is soaked into the crystals after co-crystallization with dcAdoMet were prepared as follows. Crystals of the complex of *Pf*SpdS with MTA or dcAdoMet were soaked for 30 min in the crystallization reservoir solution to which 1 m*M* MTA or dcAdoMet, 1 m*M* putrescine and 20% glycerol (for cryoprotection) were added. All other crystals containing ligands were soaked for 30 s in a cryosolution containing 20% glycerol and 1 m*M* of the corresponding ligand prior to data collection.

### 2.2. Data collection   

Data were collected on beamlines I911-2 and I911-3 at the MAX-lab synchrotron facility in Lund. Processing and scaling were performed with *XDS* (Kabsch, 2010 ▶). *Phaser* (McCoy *et al.*, 2007 ▶) was used for solving the structures by molecular replacement using the coordinates of PDB entry 2i7c (Dufe *et al.*, 2007 ▶) as a search model. The structures were refined with *REFMAC*5 (Murshudov *et al.*, 2011 ▶) and rebuilt using *Coot* (Emsley & Cowtan, 2004 ▶). Model validation was performed using the integrated validation tools in *PHENIX* (Urzhumtseva *et al.*, 2009 ▶; Adams *et al.*, 2010 ▶; Chen *et al.*, 2010 ▶).

## 3. Results   

### 3.1. Overall structures of the complexes   

**Figure 2:**
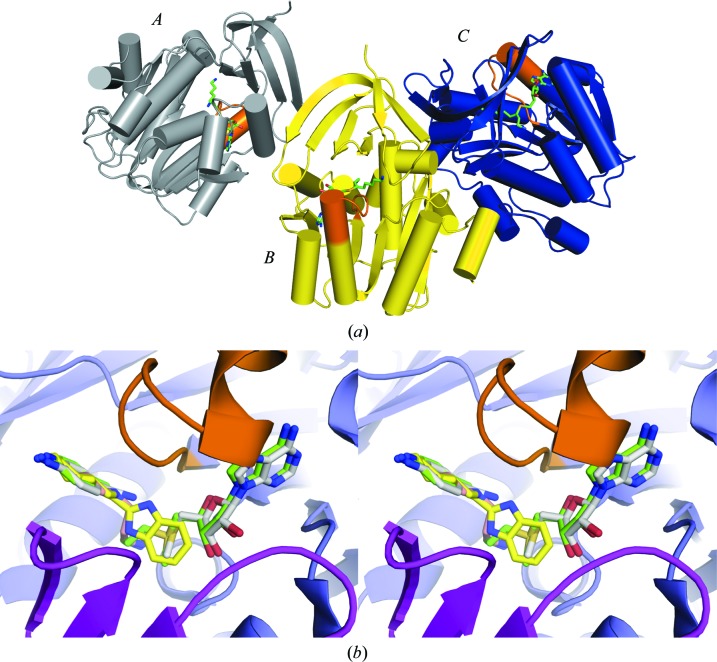
The overall structure of *Pf*SpdS and ligand binding. (*a*) The three monomers within the crystallographic asymmetric unit. A stick model of dcAdoMet (green, carbon; red, oxygen; blue, nitrogen; yellow, sulfur) is also shown to highlight the position of the active site. The gatekeeper loop (residues 196–208) is highlighted in orange. (*b*) A stereoview showing a superposition of the ligands studied in this work. C atoms of dcAdoMet are shown in green, those of MTA and 4MAN in grey, those of putrescine and spermidine in pink and those of BIPA in yellow. All figures were prepared with *PyMOL* (v1.6; Schrödinger) and chain *C* was used.

**Figure 3:**
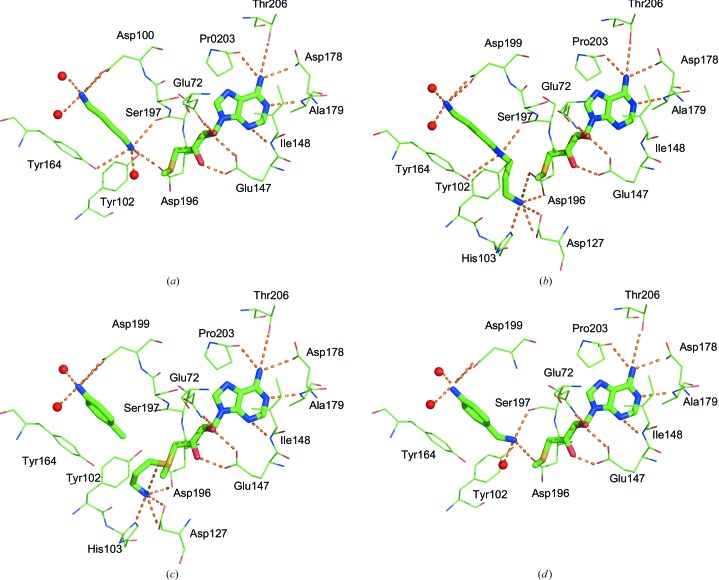
Schematic presentation of ligand binding and interactions in the active site of *Pf*SpdS. (*a*) The complex with putrescine and MTA. (*b*) The complex with spermidine and MTA. (*c*) The complex with 4MAN and dcAdoMet. (*d*) The complex with 4AMA and MTA. The amino-acid side chains and bound compounds are shown in stick representation (N atoms, blue; O atoms, red; C atoms, green; S atoms, yellow). Dashed lines represent potential hydrogen bonds.

**Figure 4:**
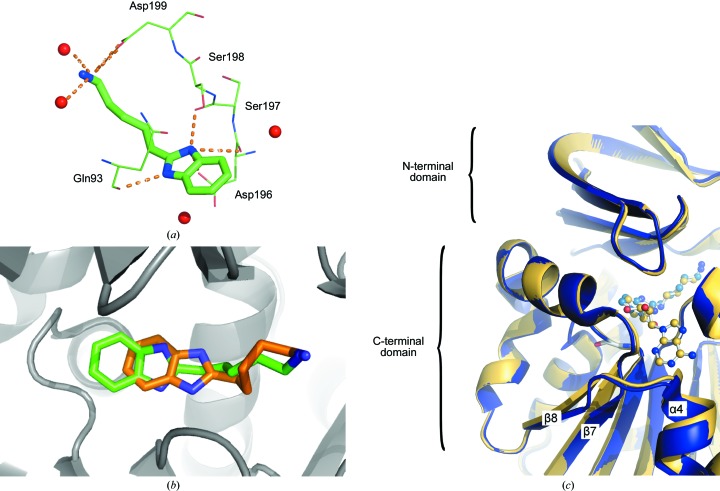
Complex of *Pf*SpdS with BIPA. (*a*) Schematic presentation of BIPA binding in the active site of *Pf*SpdS with the potential hydrogen bonds shown as dashed lines. Water molecules are shown as red spheres, N atoms in blue and O atoms in red. (*b*) A superposition of the crystal structure of BIPA (green C atoms) on the structure of the ligand obtained in docking experiments (brown C atoms; Jacobsson *et al.*, 2008 ▶), showing the shift of about 1.9 Å between the two structures. (*c*) Superposition of the structure of the complex of *Pf*SpdS with BIPA (blue) on the structure of the dcAdoMet complex, showing the slight relative shift of the C-terminal domain (by about 1 Å). dcAdoMet is shown as a ball-and-stick model (yellow C atoms).

All five complexes studied here were found to crystallize in space group *C*2 with unit-cell parameters similar to those observed in the previous work. The three monomers in the asymmetric unit are shown in Fig. 2 ▶(*a*), in which the yellow and blue monomers constitute the functional dimer of the enzyme. The buried surface area between these monomers is 1460 Å^2^, which is in the range of stable protein–protein interactions (Jones & Thornton, 1996 ▶). The third monomer constitutes part of a second dimer in which the subunits are related by the twofold symmetry axis of the crystal. Data-collection and refinement statistics are shown in Table 1 ▶ and ligand-validation statistics are shown in Table 2 ▶. The four crystal structures that contain two ligands in the active site (MTA/putrescine, MTA/spermidine, MTA/4AMA and dcAdoMet/4MAN) have 100% occupancy in all three protein molecules within the asymmetric unit (Fig. 2 ▶ *a*) and clear electron density for the gatekeeper loop. In the structure containing only BIPA, electron density for the ligand was detected in the active sites of chains *A* and *C* but not chain *B*, presumably owing to crystal contacts with the neighbouring asymmetric unit restricting the conformation of the monomer. In addition, the gatekeeper loop in chain *B* was disordered and could not be built into electron density. A stereoview showing the superposition of ligands from four of the complex structures bound to both parts of the active site is shown in Fig. 2 ▶(*b*). Details of ligand interactions are shown in Fig. 3 ▶; for the structure with only BIPA bound, stereoviews and interaction schematics are shown in Fig. 4 ▶. Simulated-annealing OMIT maps for all of the bound ligand structures are shown in Supplementary Fig. S1.

### 3.2. Complexes of *Pf*SpdS with MTA/putrescine and MTA/spermidine   

Fig. 3 ▶(*a*) shows the active site of *Pf*SpdS with bound MTA and putrescine and illustrates the network of potential hydrogen-bonding interactions involving each of the ligands. MTA is bound in the dcAdoMet part of the active site but is shifted by about 0.5 Å towards the putrescine-binding site compared with its position in the structure of the complex of human SpdS with MTA and putrescine (PDB entry 2o06; Wu *et al.*, 2007 ▶). In this position several hydrogen bonds to surrounding protein atoms are possible: between the ribose 2′ hydroxyl O atom and the side-chain carbonyl O atom of Gln72, between the adenine ring N1 atom and the amide N atom of Ala179 and between the adenine exocyclic amine N atom and a carboxylate O atom of Asp178 and/or the carbonyl O atom of the gatekeeper loop residue Pro203. Hydrophobic contacts with the adenine ring are observed with the methyl group of the gatekeeper loop residue Ala204 and between C2 and the side chain of Ile148.

The position of putrescine in the active site is also similar to that found in the human SpdS structure (PDB entry 2o06; Wu *et al.*, 2007 ▶). Putrescine binds with its proximal N atom (the one closest to dcAdoMet) located in a polar environment, with several possibilities for hydrogen-bonding interactions, including the hydroxyl O atom of Tyr102, the carboxylate and backbone carbonyl O atoms of gatekeeper residues Asp196 and Ser197, respectively, and a water molecule. The distal amino group is at a hydrogen-bonding distance from both O atoms of Asp199 (Fig. 3 ▶ *a*). This extensive network of interactions between the N-terminal part of the gatekeeper loop and putrescine and between the C-terminal part and MTA suggests that the ligands in both halves of the active site may contribute to maintaining the structured conformation of the gatekeeper loop. All of the interactions between gatekeeper loop residues and the two ligands are predicted to be maintained in SpdS from other organisms, since the specific residues involved in them are highly conserved and mutations of these residues reduce or even completely abolish enzymatic activity (Ikeguchi *et al.*, 2006 ▶; Wu *et al.*, 2007 ▶; Lee *et al.*, 2013 ▶).

When putrescine was soaked into crystals of the complex of *Pf*SpdS with dcAdoMet the crystals were not visibly altered and diffraction data were collected to 1.75 Å resolution. Analysis of the electron density shows the aminopropyl group detached from dcAdoMet and attached to the proximal N atom of putrescine, resulting in a structure of the complex with MTA and spermidine (Fig. 3 ▶ *b*). The occupancy of the two ligands is 100% in all of the molecules in the asymmetric unit, suggesting that complete conversion occurs in the crystals. No changes were observed in residue conformations around MTA or spermidine compared with the structures containing either dcAdoMet or putrescine. This is consistent with the observed stability of the crystals upon addition of putrescine. A change in the overall conformation of the protein may affect crystal contacts and result in loss of diffraction or crystal cracking.

The interactions of MTA in the structure are identical to those shown in Fig. 3 ▶(*a*) for the structure of the complex with MTA and putrescine. Spermidine binds in essentially the same orientation as putrescine (Fig. 3 ▶ *b*), with the exception of the proximal N atom, which is located within hydrogen-bonding distance of His103, Asp127 and the gatekeeper loop Asp196, mimicking the aminopropyl N atom of dcAdoMet (Fig. 3 ▶ *c*). The central secondary amine of spermidine is positioned at hydrogen-bonding distance from Ser197 and Tyr102, while a water molecule visible in Fig. 3 ▶(*a*) below the proximal N atom of putrescine has been displaced and is not detected in the structure.

### 3.3. Complexes of *Pf*SpdS with dcAdoMet/4MAN and MTA/4AMA   

Crystals of *Pf*SpdS grown in the presence of 4MAN alone or after pre-incubation of the protein with MTA followed by incubation with 4MAN showed no electron density for the ligand in the active site. On the other hand, pre-incubation of the protein with dcAdoMet followed by incubation with 4MAN resulted in crystals containing both dcAdoMet and 4MAN in the active site, suggesting that stabilization of the gatekeeper loop is required for binding of this ligand. As shown in Fig. 3 ▶(*c*), 4MAN binds in an orientation which positions its amino group at a hydrogen-bonding distance from the carboxylate O atoms of Asp199 and two water molecules. This interaction mimics the amine of putrescine and presumably contributes to maintaining the conformation of the gatekeeper loop. A π-stacking interaction can occur between Tyr264 and the aromatic ring of 4MAN, with a distance of about 4.0 Å between the ring planes; similar distances have been found for π-stacking of phenyl or phenol side chains in proteins (McGaughey *et al.*, 1998 ▶). The pose and interactions of the adenosyl and ribose moieties of dcAdoMet (Fig. 3 ▶ *c*) are very similar to those of MTA (compare Fig. 3 ▶ *a*). The aminopropyl group is located within hydrogen-bonding distance of the N^∊^ atom of His103, the carboxylate O atoms of Asp127 and the gatekeeper loop residue Asp196. These interactions are similar to those made by the proximal amino group of spermidine (Fig. 3 ▶ *b*) and to those reported in previous publications (Wu *et al.*, 2007 ▶; Dufe *et al.*, 2007 ▶). The interactions of dcAdoMet with residues at both the beginning and end of the gatekeeper loop suggest that the ligand holds the loop in place like a clamp by the formation of bonds to both of its ends.

4AMA was not among the compounds studied by Shirahata *et al.* (1991 ▶), who mostly studied methyl derivatives rather than aminomethyl derivatives such as 4AMA. Although it had very low inhibitory activity (about 15% at 1 m*M* concentration, unpublished data), 4AMA was chosen with the aim of assessing the effect of a polar group that might mimic the proximal N atom of putrescine. In contrast to 4MAN, 4AMA was found in the active site of *Pf*SpdS when the protein was pre-incubated with MTA but not with dcAdoMet. The resulting structure shows 4AMA bound essentially in the same fashion as 4MAN (Fig. 3 ▶ *d*), with the exception of the aminomethyl group, which as predicted forms interactions reminiscent of those formed by the proximal putrescine N atom. As shown in the figure, the aminomethyl N atom is positioned at a hydrogen-bonding distance from the carboxylate O atoms of Asp196, the backbone carbonyl O atom of Ser197 and a water molecule. However, in this case the distances to the hydroxyl O atom of Tyr102 are longer (∼4 Å) than the respective distance observed for the putrescine N atom (3.2 Å). In this position, the aminomethyl N atom of 4AMA would make a steric clash with the aminopropyl group of bound dcAdoMet, which may explain the low inhibitory activity of 4AMA. The aniline N atom is positioned within hydrogen-bonding distance of the carboxylate O atoms of Asp199 and two water molecules, as in the complex with 4MAN. The aromatic ring can also engage in a π-stacking interaction with Tyr264, with a ring centre-to-centre distance of 4.0 Å. The binding mode of MTA was essentially identical to those found in the other structures.

### 3.4. Complex with BIPA   

In contrast to 4MAN and 4AMA, co-crystallization of BIPA with the protein did not require the presence of dcAdoMet or MTA, thus defining a novel ligand-binding mode. The orientation of BIPA in the active site relative to the other ligands described above is shown in Fig. 2 ▶(*b*). Fig. 4 ▶(*a*) shows the interactions made by BIPA with neighbouring amino-acid residues. The compound is bound in the active sites of chains *A* and *C* only (Fig. 2 ▶ *a*), presumably as a result of crystal contacts of chain *B* with the neighbouring asymmetric unit. The gatekeeper loop is disordered in chain *B*, reflecting its empty ligand-binding site. As shown in Fig. 4 ▶(*b*), the orientation of the ligand is similar to the orientation predicted in the *in silico* screening study by Jacobsson *et al.* (2008 ▶), although its position was shifted by about 1.9 Å relative to the position in the predicted structure. Despite the different scaffold, the benzimidazole moiety of BIPA occupies the binding site of the dcAdoMet aminopropyl group. One of the ring N atoms is at a hydrogen-bonding distance from the backbone carbonyl O atom of Gln93 (Fig. 4 ▶ *a*), while the aminopentyl group is positioned in the putrescine-binding site, which gives the terminal amino N atom the opportunity to interact with the carboxylate O atoms of Asp199 and two water molecules, thus mimicking the interactions formed by putrescine.

Although BIPA has no inhibitory activity at 1 m*M* (unpublished data), it is unique among the ligands studied to date in crystallizing with *Pf*SpdS when bound alone, despite anchoring only the N-terminal part of the gatekeeper loop. The remaining part of the loop has a conformation similar to that found in structures with ligands bound to both parts of the active site. BIPA binding correlates with some minor structural changes near the active site: helix α4 and the C-terminal parts of β-strands β7 and β8 of the C-terminal domain are shifted away by about 1 Å away from the N-terminal domain compared with structures containing two ligands in the active site (Fig. 4 ▶ *c*). This shift may be a result of the absence of interactions between BIPA and the C-terminal part of the gatekeeper loop. It also suggests that the conformation of the gatekeeper loop can be sensed by the N- and C-terminal domains, implying that the loop may play a role in the communication between the two domains of SpdS. Despite the low inhibitory activity of BIPA, its binding mode may still be considered in the design of new inhibitors.

## 4. Discussion   

This work presents the structures of *Pf*SpdS in complex with three different ligands, 4MAN, 4AMA and BIPA, of which 4MAN has verified inhibitory activity. In addition, we present the complexes with the substrate putrescine and the reaction product MTA, and with the reaction products MTA and spermidine. The latter was obtained as a result of the enzymatic reaction taking place when putrescine was soaked into crystals of the complex of SpdS with dcAdoMet. The structures of the complexes stress the importance of earlier observations (Korolev *et al.*, 2002 ▶; Dufe *et al.*, 2007 ▶; Wu *et al.*, 2007 ▶) that had suggested that ligand binding to the putrescine site of SpdS requires stabilization of the conformation of the gatekeeper loop by a ligand present in the dcAdoMet site. On the other hand, the complex with BIPA shows that ligands with certain scaffolds may bind alone to the enzyme and still form the interactions required for loop stabilization.

Based on the results of this study and earlier data (Shira­hata *et al.*, 1991 ▶; Haider *et al.*, 2005 ▶; Dufe *et al.*, 2007 ▶), a classification of potential *Pf*SpdS ligands into two groups can be proposed. Group 1 contains ligands that may stabilize the flexible loop and do not require the presence of another ligand to bind to the enzyme. Compounds that belong to this group include (1a) the substrate dcAdoMet, the reaction product MTA and their analogues (Hibasami *et al.*, 1980 ▶; Lakanen *et al.*, 1995 ▶; Šečkutė *et al.*, 2011 ▶), a representative of which is the so-called multi-substrate adduct inhibitor AdoDATO (Tang *et al.*, 1980 ▶) that combines the scaffolds of MTA and dcAdoMet (Fig. 1 ▶ *b*), and (1b) ligands that bind to the free form of the enzyme similarly to BIPA employing an alternative gatekeeper-loop stabilization mechanism. Group 2 contains compounds such as 4MAN, 4AMA and 4MCHA, which bind to the putrescine site only in the presence of a gatekeeper loop-stabilizing ligand in the dcAdoMet site.

While 4MAN and 4MCHA are moderate inhibitors of SpdS, 4AMA has a much weaker inhibitory activity (around 15% at 1 m*M* concentration). The primary value of these compounds is in their binding mode, which shows that aromatic groups and not only linear chains may be used in the design of competitors of putrescine, a finding that should be considered in the design of new inhibitors of SpdS. Additional studies are required to explore the details of SpdS inhibition by these compounds, for example to find out whether their inhibitory effect depends solely on competition with putrescine or includes some additional factors that would affect the binding of dcAdoMet or MTA. The structure of the MTA–putrescine complex (Fig. 3 ▶ *a*) suggests that putrescine may directly bind to the enzyme after the release of spermidine and prior to MTA/dcAdoMet exchange. The structure also suggests that even putrescine-competitive inhibitors may bind at this stage of the enzymatic reaction.

Although enzyme-activity studies show that MTA and a number of its analogues are generally weak inhibitors of SpdS (around 30% inhibition at a concentration of 0.1 m*M*), SpmS is inhibited with a much higher potency (Pajula & Raina, 1979 ▶; Hibasami *et al.*, 1980 ▶; Ikeguchi *et al.*, 2006 ▶). Wu *et al.* (2008 ▶) have discussed the rationale for the differences in MTA binding by SpdS and SpmS. Nonetheless, it has been shown that increasing MTA concentrations in the cell may still reduce the activity of SpdS (Pegg & Michael, 2010 ▶). Based on this, it has been argued, for example, that the effect of MTA phosphorylase (MTAP) inhibitors on cancer cells could be the result of inhibition of SpmS by elevated MTA levels (Basu *et al.*, 2011 ▶). However, it was subsequently shown that full inhibition of MTAP in lung cancer cells had no effect on polyamine levels (Basu *et al.*, 2011 ▶). Nevertheless, it would be of interest to investigate whether changes in the MTA and dcAdoMet levels in *Plasmodium* would affect spermidine and spermine levels as a result of inhibition of *Pf*SpdS by MTA and putrescine. For example, a combined inhibition of MTAP and AdoMetDC may disturb the intracellular dcAdoMet/MTA balance, which would allow MTA to be an efficient competitor with dcAdoMet. In addition, putrescine or even another ligand bound at the putrescine site could contribute to enzyme inhibition by stabilizing the gatekeeper loop, which could result in more stable MTA binding. Notably, in the structures of the complexes of human SpdS with MTA, MTA/putrescine and MTA/spermidine the average *B* factors for the gatekeeper loop were found to be around 42, 28 and 29 Å^2^, respectively, clearly showing higher flexibility of the loop when MTA is bound alone. A similar picture is seen in the complexes with *Pf*SpdS: the complex with MTA (PDB entry 2hte; Vedadi *et al.*, 2007 ▶) has an average *B* factor of 32.5 Å^2^, whereas the complexes with MTA/putrescine (PDB entry 4bp1) and MTA/spermidine (PDB entry 4cxm) have average *B* factors of 21.9 and 21.4 Å^2^, respectively.

Analysis of earlier structures and the structures presented here shows that the interactions of bound ligands with both the N- and C-terminal ends of the flexible loop are essential for loop stabilization. Thus, in the complex with bound dcAdoMet and 4MAN these interactions involve the exocyclic amine of the adenine moiety and the aminopropyl group of dcAdoMet with gatekeeper-loop residues Pro203 and Asp196, respectively (Fig. 3 ▶ *c*), while the amino group of the inhibitor 4MAN occupies the position of the distal amino group of putrescine and may contribute a hydrogen bond to Asp199. Of these three residues, Asp196 and Asp199 are invariant in SpdS sequences, while Pro203 is highly conserved. It has previously been suggested (Wu *et al.*, 2007 ▶) that replacement of the residue corresponding to Pro203 by glutamine was one of the factors that accounted for a higher flexibility of the gatekeeper loop in *T. maritima* SpdS (Glu178 in *T. maritima* numbering). In *E. coli* the replacement of the corresponding residue (Pro165) by glutamine resulted in an enzyme that had around 50% activity compared with the wild-type enzyme (Lee *et al.*, 2013 ▶). When MTA is bound in the complex with putrescine, the proximal amino group of putrescine takes over the interactions of the aminopropyl group of dcAdoMet with Asp196 (Fig. 3 ▶ *a*). A similar arrangement is observed when MTA and spermidine are bound in the active site (Fig. 3 ▶ *b*). Thus, one of the consequences of the conversion of dcAdoMet to MTA is the lost interaction with Asp196, which presumably leads to the lower affinity of MTA with compared with dcAdoMet and which together with the higher flexibility of the gatekeeper loop may trigger MTA/dcAdoMet exchange.

The binding mode of BIPA, on the other hand, enables a different manner of loop stabilization. Thus, one amino group of the imidazole ring appears to stabilize the N-terminal part of the gatekeeper loop by forming a hydrogen bond to Asp196, although the backbone carbonyls of Ser197 and Ser198 and the side chain of Ser198 are also at a hydrogen-bonding distance. The absence of interactions with Pro203 at the other end of the loop may be one of the reasons that BIPA is a poor inhibitor of SpdS. However, the binding mode of BIPA can still be used in future efforts to design new potent inhibitors of SpdS.

The results of this work indicate that the interplay between ligands bound to both parts of the active site and their interactions with both ends of the flexible loop are essential in controlling ligand binding to the active site of SpdS. In other words, the gatekeeper loop is not a passive structural element, but rather an active player in controlling not only ligand binding but also the enzymatic reaction. These findings need to be taken into account in any future strategy for the design of new inhibitory compounds for SpdS.

## Supplementary Material

PDB reference: spermidine synthase, complex with dcAdoMet and 4MAN, 4bp3

PDB reference: complex with MTA and putrescine, 4bp1

PDB reference: complex with MTA and 4AMA, 4uoe

PDB reference: complex with MTA and spermidine, 4cxm

PDB reference: complex with BIPA, 4cwa

Supporting Information.. DOI: 10.1107/S1399004714027011/kw5110sup1.pdf

## Figures and Tables

**Table 1:** Data-collection and refinement statistics Values in parentheses are for the highest resolution shell.

	MTA + putrescine	MTA + spermidine	dcAdoMet + 4MAN	MTA + 4AMA	BIPA
PDB code	4bp1	4cxm	4bp3	4oue	4cwa
Data collection					
Source	MAX-lab I911-2	MAX-lab I911-3	MAX-lab I911-2	MAX-lab I911-2	MAX-lab I911-2
Space group	*C*121	*C*121	*C*121	*C*121	*C*121
Unit-cell parameters					
*a* ()	200.75	197.90	195.46	198.05	197.50
*b* ()	34.97	134.40	132.79	135.62	134.38
*c* ()	48.60	48.30	49.16	48.31	148.28
()	96.60	95.50	94.86	95.33	94.53
Completeness (%)	99.6 (98.7)	98.6 (98.7)	93.7 (78.5)	96.0 (94.9)	97.9 (94.2)
Resolution range ()	28.652.17 (2.312.17)	44.941.75 (1.861.75)	28.651.76 (1.861.76)	29.542.05 (2.102.05)	26.632.02 (2.142.02)
*R*_merge_	0.12 (0.67)	0.05 (0.59)	0.05 (0.39)	0.07 (0.48)	0.09 (0.56)
*R*_meas_	0.142	0.065	0.062	0.088	0.070
*I*/(*I*)	11.0 (2.2)	15.0 (2.3)	15.4 (2.4)	14.2 (3.2)	13.2 (3.7)
No. of unique reflections	66992	124670	117262	76131	80736
No. of accepted reflections	228987	344231	405430	247138	255888
Wilson *B* factor (A^2^)	22.5	20.7	19.6	22.6	19.0
CC_1/2_	0.994 (0.705)	0.999 (0.750)	0.998 (0.872)	0.998 (0.873)	0.998 (0.925)
CC*	0.999 (0.909)	1.000 (0.926)	1.000 (0.965)	0.999 (0.966)	0.999 (0.980)
Refinement					
Resolution range ()	28.652.17	44.941.75	28.651.76	29.532.05	26.632.02
*R*_model_	0.187	0.188	0.188	0.199	0.213
*R*_free_	0.234	0.222	0.219	0.245	0.244
Test-set size (%)	5.10	5.28	5.25	5.29	5.26
No. of protein residues	842	848	842	841	839
No. of water molecules	410	496	391	426	352
Bound ligands	3 MTA, 3 putrescine, 1 glycerol, 1 PEG	3 MTA, 3 spermidine	3 dcAdoMet, 3 4MAN, 1 glycerol, 2 PEG	3 MTA, 3 4AMA, 2 glycerol, 1 PEG	2 BIPA, 1 PEG
Average *B* factor (^2^)	28.1	25.0	23.4	28.0	27.0
Clash score	3.6	3.3	6.1	3.2	2.9
Rotamer outliers	29 [4%]	19 [2%]	20 [3%]	14 [2%]	13 [2%]
Model geometry (r.m.s. deviations from ideal geometry†)					
Bond lengths ()	0.021	0.025	0.025	0.021	0.021
Bond angles ()	2.03	2.10	2.43	1.92	1.93
Ramachandran plot					
Most favoured (%)	95.0	97.0	97.0	97.0	96.8
Additional allowed (%)	4.6	2.9	3.0	3.0	3.2
Disallowed (%)	0.4 [chain *A*, Ile235; chain *B*, Ser69, Glu219]	0.1 [chain *A*, Ile235]	0.0	0.0	0.0

† Ideal geometry for proteins (Engh Huber, 1991 ▶).

**Table 2:** Ligand-validation statistics

PDB code	Ligand	Chain	R.m.s.d.† ()	RSCC‡	LLDF§	*B*_iso_ § (^2^)
4bp1	MTA	*A *	*AB*: 0.25	0.98	0.9	23
		*B*	*BC*: 0.24	0.98	0.6	22
		*C*	*AC*: 0.24	0.98	0.2	19
	Putrescine	*A *	*AB*: 0.48	0.91	5.0	44
		*B*	*BC*: 0.98	0.97	3.9	25
		*C*	*AC*: 1.01	0.98	3.7	23
4cxm	MTA	*A *	*AB*: 0.20	0.99	0.9	22
		*B *	*BC*: 0.12	0.98	0.6	20
		*C*	*AC*: 0.25	0.98	1.0	17
	Spermidine	*A *	*AB*: 0.84	0.94	1.6	34
		*B *	*BC*: 0.91	0.94	4.3	28
		*C*	*AC*: 0.25	0.96	2.6	23
4bp3	dcAdoMet	*A *	*AB*: 0.21	0.96	0.2	25
		*B *	*BC*: 0.20	0.96	0.9	22
		*C*	*AC*: 0.16	0.96	0.3	20
	4MAN	*A *	*AB*: 0.10	0.96	0.5	26
		*B *	*BC*: 0.18	0.99	0.6	18
		*C*	*AC*: 0.21	0.98	0.8	15
4uoe	MTA	*A *	*AB*: 0.24	0.97	0.3	21
		*B*	*BC*: 0.24	0.96	0.5	22
		*C*	*AC*: 0.18	0.98	0.1	23
	4AMA	*A*	*AB*: 0.29	0.93	0.6	37
		*B*	*BC*: 0.32	0.96	0.9	21
		*C*	*AC*: 0.27	0.98	0.8	19
4cwa	BIPA	*A *	*AB*:	0.88	2.7	41
		*B*	*BC*:		No ligand	
		*C*	*AC*: 0.44	0.93	3.9	26

† R.m.s.d. calculated between corresponding ligand atoms in superimposed protein chains *A*, *B* and *C*.

‡ Values from validation in *PHENIX* (Adams *et al.*, 2010 ▶).

§ Values taken from the PDB validation report.
