# Supplementary material for: Three-dimensional structures of Plasmodium falciparum spermidine synthase with bound inhibitors suggest new strategies for drug design
Source: Acta Crystallogr D Biol Crystallogr. 2015 Feb 26;71(Pt 3):484–93. doi: 10.1107/S1399004714027011 (PMC4356361; doi:10.1107/S1399004714027011)
Supplement: Supplementary file 1 [file d-71-00484-sup1.pdf]

BIOLOGICAL
CRYSTALLOGRAPHY

Volume 71 (2015)

Supporting information for article:

Three-dimensional structures of *Plasmodium falciparum* spermidine synthase with bound inhibitors suggest new strategies for drug design

Janina Sprenger, Bo Svensson, Jenny Hålander, Jannette Carey, Lo Persson and Salam Al-Karadaghi

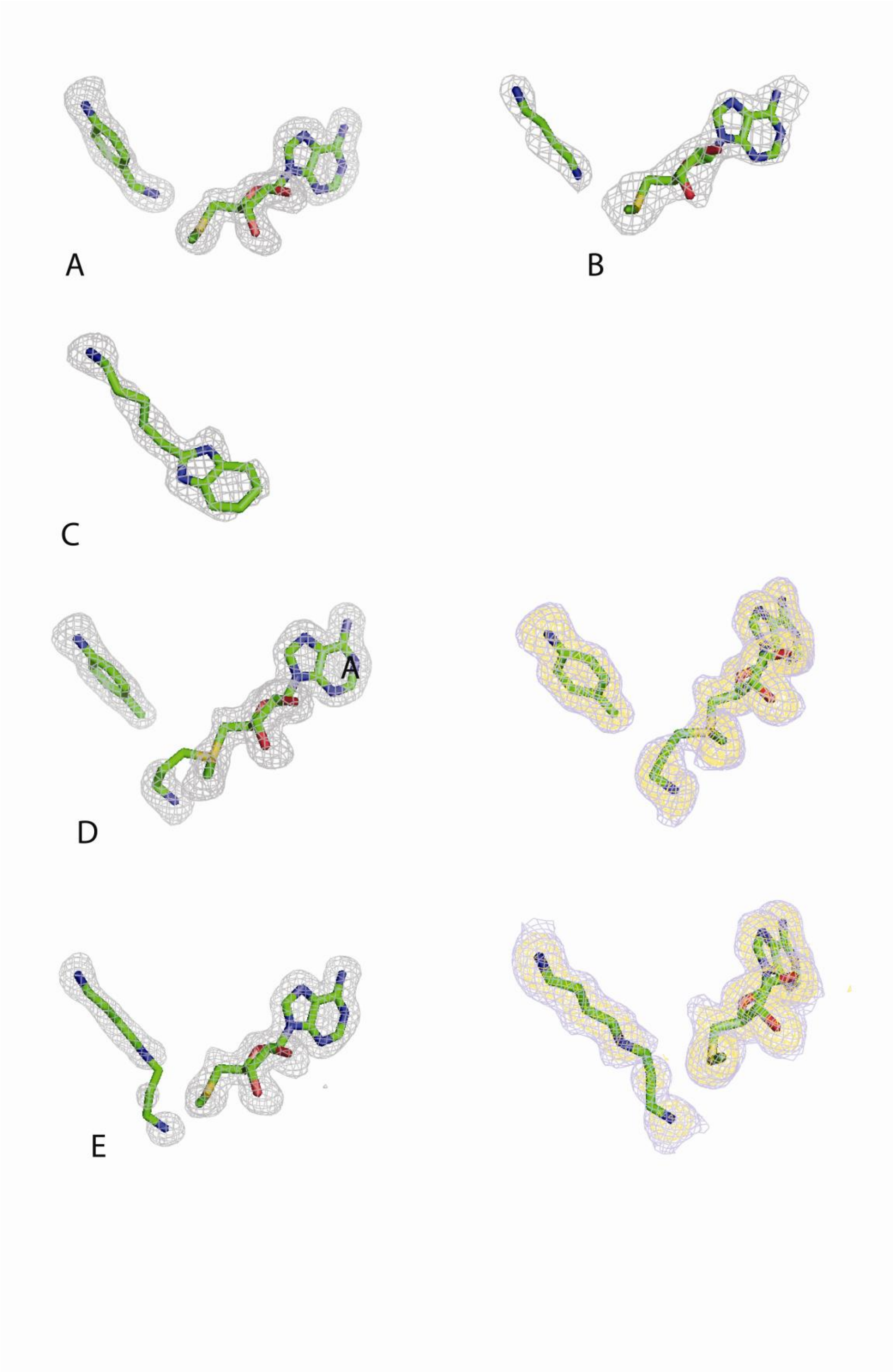

Figure S1 Simulated annealing Fo-Fc OMIT maps of ligands on complex with *Pf*SpdS**.** The simulated annealing Fo-Fc ligand OMIT maps are shown as grey mesh at 3σ, carve=2 Å.

The ligands are presented as stick models (nitrogen atoms blue, oxygen red, carbon green, and sulfur yellow). Chain C is used in all cases: **A.** 4AMA:MTA **B.** Putrescine:MTA **C.** BIPA **D.** 4MAN:dcAdoMet. To better visualize the electron density around the flexible aminopropyl group the maps are shown at different σ levels. The left figure shows the Fo-Fc ligand OMIT map as grey mesh at 3σ; in the right figure grey mesh is drown at 2.5σ and yellow mesh at 3σ **E.** Spermidine:MTA The counter level for the maps in the left and right figures as described above for figure D.
